# The Efficiency of Hybrid Intelligent Models in Predicting Fiber-Reinforced Polymer Concrete Interfacial-Bond Strength

**DOI:** 10.3390/ma15093019

**Published:** 2022-04-21

**Authors:** Mohammad Sadegh Barkhordari, Danial Jahed Armaghani, Mohanad Muayad Sabri Sabri, Dmitrii Vladimirovich Ulrikh, Mahmood Ahmad

**Affiliations:** 1 Department of Civil and Environmental Engineering, Amirkabir University of Technology, Tehran 1591634311, Iran; m.s.barkhordari@aut.ac.ir; 2 Department of Urban Planning, Engineering Networks and Systems, Institute of Architecture and Construction, South Ural State University, 76, Lenin Prospect, 454080 Chelyabinsk, Russia; ulrikhdv@susu.ru; 3 Peter the Great St. Petersburg Polytechnic University, 195251 St. Petersburg, Russia; 4 Department of Civil Engineering, University of Engineering and Technology Peshawar (Bannu Campus), Bannu 28100, Pakistan; ahmadm@uetpeshawar.edu.pk

**Keywords:** fiber-reinforced polymer, interfacial bond, hybrid algorithm, neural network, machine learning

## Abstract

Fiber-reinforced polymer (FRP) has several benefits, in addition to excellent tensile strength and low self-weight, including corrosion resistance, high durability, and easy construction, making it among the most optimum options for concrete structure restoration. The bond behavior of the FRP-concrete (FRPC) interface, on the other hand, is extremely intricate, making the bond strength challenging to estimate. As a result, a robust modeling framework is necessary. In this paper, data-driven hybrid models are developed by combining state-of-the-art population-based algorithms (bald eagle search (BES), dynamic fitness distance balance-manta ray foraging optimization (dFDB-MRFO), RUNge Kutta optimizer (RUN)) and artificial neural networks (ANN) named “BES-ANN”, “dFDB-MRFO -ANN”, and “RUN-ANN” to estimate the FRPC interfacial-bond strength accurately. The efficacy of these models in predicting bond strength is examined using an extensive database of 969 experimental samples. Compared to the BES-ANN and dFDB-MRFO models, the RUN-ANN model better estimates the interfacial-bond strength. In addition, the SHapley Additive Explanations (SHAP) approach is used to help interpret the best model and examine how the features influence the model’s outcome. Among the studied hybrid models, the RUN-ANN algorithm is the most accurate model with the highest coefficient of determination (R^2^ = 92%), least mean absolute error (0.078), and least coefficient of variation (18.6%). The RUN-ANN algorithm also outperformed mechanics-based models. Based on SHAP and sensitivity analysis method, the FRP bond length and width contribute more to the final prediction results.

## 1. Introduction

Fiber-reinforced polymer (FRP) has been widely employed as a successful approach for reinforcing concrete structures [1,2,3] and steel structures [4,5]. FRP comprises numerous layers, and essential performance advantages, such as excellent mechanical properties, fatigue, creep, and corrosion resistance [6,7]. According to relevant studies, FRP composite is a potential steel substitute due to its lightweight, high strength, and improved fatigue and corrosion resistance [8]. A considerable amount of research effort has been allotted to investigate the bearing performance of FRP reinforced concrete structures under static and dynamic conditions. For example, Bagheri et al. [9] performed seismic analyses to obtain the optimum length of the FRP on the retrofitted columns of reinforced concrete frames. Various bond strength models based on empirical or semi-empirical methodologies have been developed [10,11,12,13,14]. Previous research has primarily relied on experimental findings from the single-lap shear test to develop bond strength formulations [15,16]. Since the valuable bond length of the FRP was not taken into account, the prediction powers of some models were limited [17,18,19].

On the other hand, most empirical frameworks may yield considerable results when applied to new data because they are generated based on the available experimental results. Furthermore, some simplified hypotheses must be used to construct these empirical strength models. Machine learning (ML), on the other hand, is one of the six branches of artificial intelligence (AI) that allows us to train machines how to complete tasks by showing them how they should be performed [20]. Several studies have recently been reported on machine learning algorithms for damage estimation and prediction in civil engineering. Based on the machine learning prediction outcomes, these studies can be divided into two groups [21,22]: (1) techniques based on regression, when the output is a continuous quantity (e.g., shear force), and (2) approaches based on classification, where the output is a discrete variable (e.g., failure mode). These algorithms can account for complex underlying interactions among input and output parameters. There is some research on the interfacial bond strength of predicting FRP-concrete (FRPC) using machine learning models [23,24,25]. Su et al. [26] used three distinct ML approaches, such as a support vector machine (SVM) and a multiple linear regression (MLR), to establish the link between the influential variables and the bond strength. They utilized random and grid search to determine the best hyperparameters for the models. Chen et al. [27] developed an FRPC interfacial bond strength prediction model built on 520 tested samples using an ensemble learning approach called gradient boosted regression trees. Basaran et al. [28] employed ML techniques, such as Gaussian process regression, artificial neural networks (ANN), SVM regression, regression tree, and multiple linear regression for FRPC bond strength prediction and compared them with experimental results. Zhou et al. [29] built artificial neural networks for estimating fiber-reinforced polymer-concrete interfacial bond strength using the backpropagation neural network method. The carbon FRP steel interfacial bond strength is predicted by Chen et al. [30] using gradient enhanced decision trees and random forests. They used 113 carbon FRP steel single-shear test samples to train the data-driven model. While modern ML-based frameworks in structural engineering problems have produced good results, new hybrid intelligent models (ANN with optimization algorithm) for predicting FRPC interfacial bond strength have yet to be discovered.

The feed-forward neural network, which is trained using backpropagation, is the most popular of the ANNs, but they have a variety of drawbacks, such as falling into local minima and learning at a slow rate [31]. According to the literature, such flaws can be solved by employing effective optimization strategies [32]. As a result, the research reported in this paper aims to address the following issues: (1) providing an accurate and efficient machine learning model for estimating the interfacial bonding strength of FRPC using hybrid approaches; (2) Examining the forecasting accuracy of the best machine learning model against that of existing mechanics-based models and (3) using the SHapley Additive exPlanation approach [33] to describe the importance and participation of input variables that influence FRPC interfacial bond strength. Hybrid algorithms are created by combining state-of-the-art optimization algorithms, such as bald eagle search (BES), dynamic fitness distance balance (dFDB)-manta ray foraging optimization (MRFO) dFDB-MRFO, and RUNge Kutta optimizer (RUN) with AAN to achieve this purposes.

## 2. Data of Single-Lap Shear Tests

**Figure 1:**
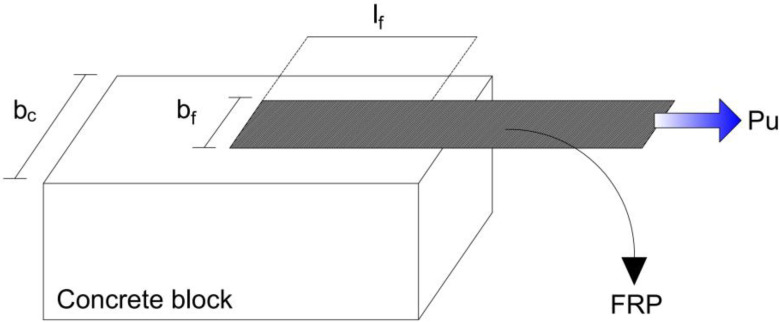
Shear deboning behavior—single-lap shear test.

Nine hundred sixty-nine test data from available single-lap shear experiments (Figure 1) on FRPC interfacial bond strength have been employed to build appropriate hybrid ML models. This laboratory information was collected by Zhou et al. [29] from available studies [13,14,34,35,36,37,38,39,40,41,42]. The design parameters or input variables retrieved from the gathered specimens are used in machine learning models. However, while establishing input parameters, parameters with no practical value for practicing engineers and parameters without meaning for existing structures were not considered, such as loading rate. Besides the test results, the general form of many existing bond strength models based on either the effective bond length model or fracture mechanics was reviewed by Zhou et al. [29] when input parameters were determined.

**Figure 2:**
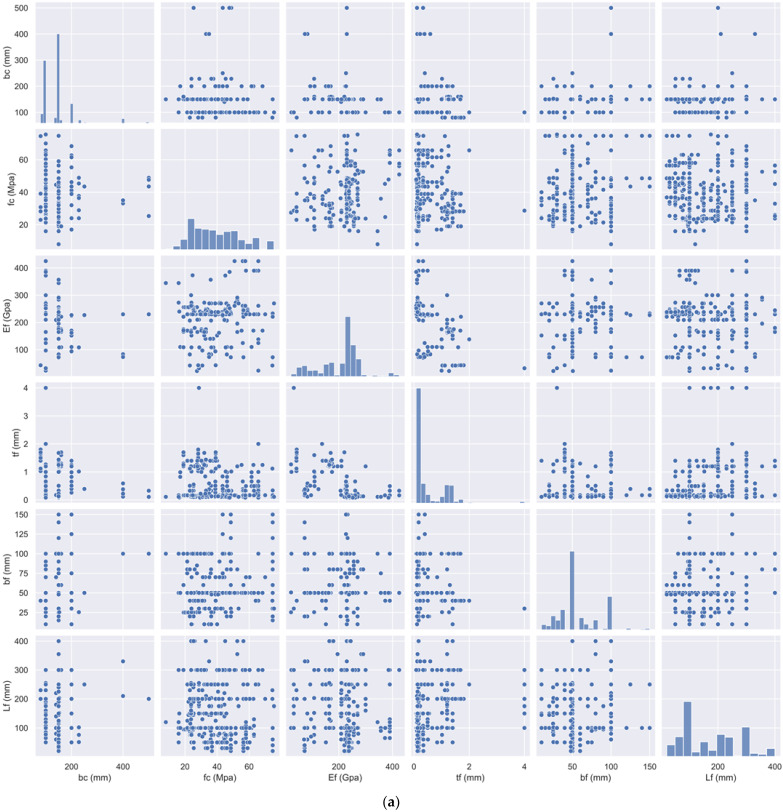

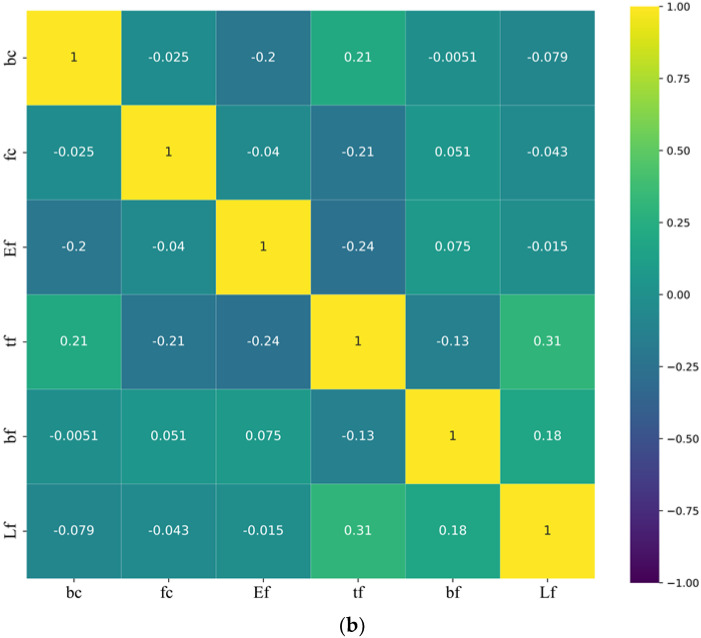
Correlation matrix and statistical distributions of input variables. (**a**) Distributions of input variables. (**b**) Correlation matrix for input variables.

Here, six design criteria are considered as input variables in these tests, and they are divided into two groups; (1) geometric dimensions: the fiber-reinforced polymer sheet (FRPS) thickness ($t_{f}$), FRPS width ($b_{f}$), the bond length of the FRPS ($l_{f}$), and width of the concrete ($b_{c}$); (2) material properties: concrete compressive strength ($f_{c}^{\prime }$) and FRPS elastic modulus ($E_{f}$). $P_{u}$ shown in Figure 1 is the ultimate bond strength of a sample. The experimental dataset is randomly divided into a testing set (20%) and a training set (80%) for the testing and training phase. To enhance the robustness of the database, the original repository has been processed according to the basic rules: (1) One sample should be eliminated from a set with the same test parameters if the ultimate bond strength differs by more than fifteen percent from the other test data and the other data points differ by less than fifteen percent. (2) If the difference between two samples in a set of data under the identical test parameters is greater than fifteen percent, the entire set of data must be discarded. The 969 samples were lowered to 855 using the filtering procedures outlined above. Table 1 presents the statistical properties of the dataset. Figure 2a shows statistical distributions of input variables. The correlation coefficient for the inputs is shown in Figure 2b. The degree of the relationship between two separate input variables is shown by each matrix element. Correlations have a very low absolute value. As a result, Multicollinearity is not a problem in this case.

## 3. Artificial Neural Network

Neurons or nodes generate the architecture of ANNs. Multiple inputs are available to each neuron, and these inputs are mixed, and after processing, the result of that combination is an output. The nodes are linked together, with each cell’s output serving as the input for the next cell. Additionally, hidden layers are the layers between the input and output layers. The connections between the neurons are weighted, reflecting the contribution amplitude of each neuron to the neurons in the adjacent layer to which it is connected. When the network object is built, the weights are randomized at first, but they are modified when the ANN is trained. Here, to enhance the predictive accuracy of ANNs, the optimization algorithms are applied to fine-tune the weights and biases of the ANN model.

### 3.1. Overview of BES-ANN Algorithm

**Figure 3:**
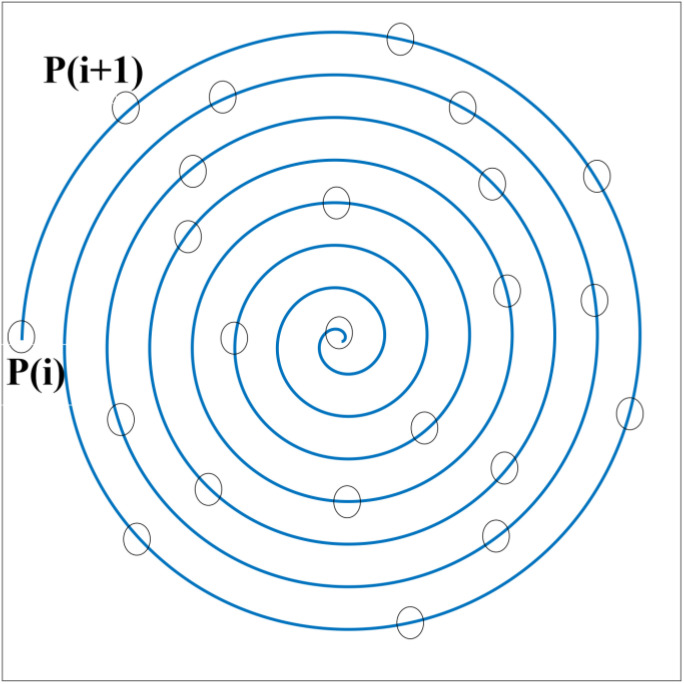
Searching within a spiral space.

**Figure 4:**
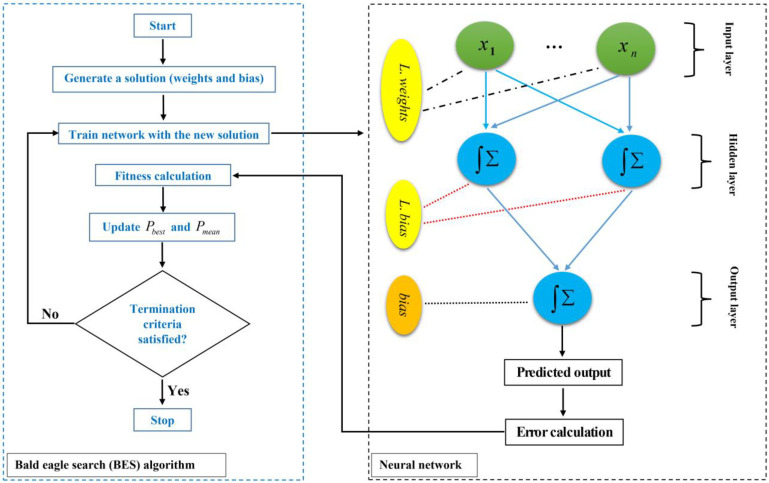
Flowchart of the BES-ANN algorithm.

The bald eagle search (BES) algorithm [43] simulates the behavior of bald eagles while hunting. As a result, this algorithm has three stages: picking the search space, searching inside the selected search area, and swooping. Within the designated search zone, bald eagles identify and select the ideal place (in terms of volume of food) where they can seek prey during the select stage. Equation (1) is a mathematical representation of this behavior.

(1) $$p(i+1)=p_{best}+\alpha \cdot r\cdot (p_{mean}-p(i))$$

where *r* is a random value in (0,1), α is a constant with a value of 2, $p_{best}$ is the best position based on previous experience, $p_{mean}$ denotes that the eagles have consumed all of the data from the previous points, and p(i) is the position at iteration *i*th. Bald eagles look for prey within the designated search space and travel in various directions inside a spiral zone (Figure 3) to speed up their search in the search phase. The best swoop location is represented mathematically in Equation (2).

(2) $$\begin{array}{l} p(i+1)=p(i)+y(i)\cdot (p(i)-p(i+1))+x(i)\cdot (p(i)-p_{mean})\\ x(i)=\frac{xr(i)}{\max(|xr|)}\;,\;y(i)=\frac{yr(i)}{\max(|yr|)}\\ xr(i)=r(i)\cdot \sin(\theta (i))\;,\;yr(i)=r(i)\cdot \cos(\theta (i))\\ \theta (i)=5\pi \cdot rand\;,\;r(i)=\theta (i)+2\cdot rand \end{array}$$

where rand is a random number in (0,1]. The flowchart of the BES-ANN algorithm is shown in Figure 4.

### 3.2. Overview of dFDB-MRFO-ANN Algorithm

**Figure 5:**
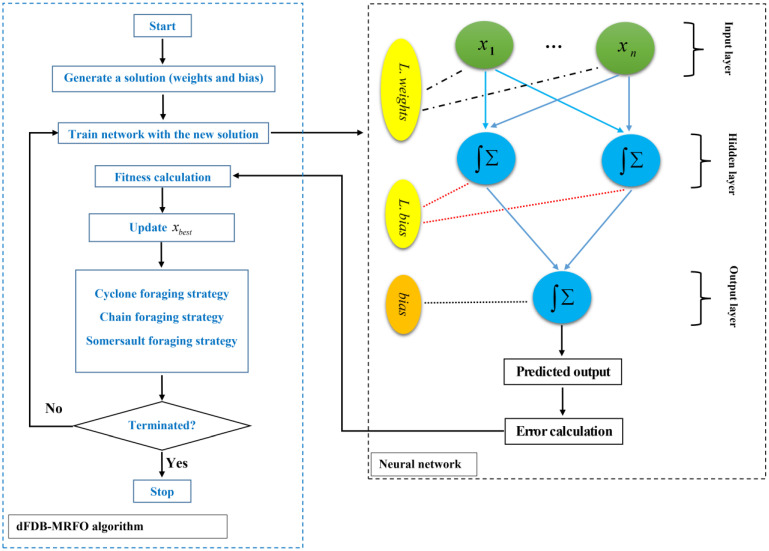
Flowchart of the dFDB-MRFO-ANN algorithm.

A Manta ray foraging algorithm is a bio-inspired method that differs from other meta-heuristic search (MHS) techniques due to the nutritional tactics that are important in determining the life cycle of the search process. Manta ray foraging optimization (MRFO) uses a powerful selection method, so-called dynamic fitness-distance balance (dFDB), in its design called dFDB-MRFO [44]. The dFDB features a dynamically changing weight parameter, which is the most essential characteristic of the dFDB algorithm. Consequently, the system is now capable of adjusting quickly and effectively to the search spaces for a variety of situations. The dFDB-MRFO has three powerful nutritional strategies [44]: (1) chain foraging: all of the manta rays are lined up. Those behind manta rays can, therefore, harvest plankton that the front manta ray cannot (Equation (3)); (2) cyclone foraging: Manta rays swim in deep water in a spiral shape toward plankton (Equation (4)); (3) somersault foraging: A food source’s location is chosen as a pivot point. Manta rays try to improve their position by rotating around this pivot (Equation (5)).

(3) $$\begin{array}{l} x_{i}^{d}(t+1)=\left\{\begin{array}{l} x_{i}^{d}(t)+r\cdot (x_{best}^{d}-x_{i}^{d}(t))+\alpha \cdot (x_{best}^{d}-x_{i}^{d}(t)),\;i=1\\ x_{i}^{d}(t)+r\cdot (x_{i-1}^{d}(t)-x_{i}^{d}(t))+\alpha \cdot (x_{best}^{d}-x_{i}^{d}(t)),\;i=2,\ldots ,N \end{array}\right.\\ \alpha =2\cdot r\cdot \sqrt{\left|\log(r)\right|} \end{array}$$

(4) $$\begin{array}{l} x_{i}^{d}(t+1)=\left\{\begin{array}{l} x_{best}^{d}+r\cdot (x_{best}^{d}-x_{i}^{d}(t))+\beta \cdot (x_{best}^{d}-x_{i}^{d}(t))\;,\;i=1\\ x_{best}^{d}+r\cdot (x_{i-1}^{d}(t)-x_{i}^{d}(t)+\beta \cdot (x_{best}^{d}-x_{i}^{d}(t))\;,\;i=2,\ldots ,N \end{array}\right.\\ \beta =2e^{r_{1}\frac{T-t+1}{T}}\cdot \sin(2\pi r_{1}) \end{array}$$

(5) $$x_{i}^{d}(t+1)=x_{i}^{d}(t)+2\cdot (r_{2}\cdot x_{best}^{d}-r_{3}\cdot x_{i}^{d}(t)),\;i=1,2,\ldots ,N$$

where $x_{i}^{d}(t)$ is a vector of a position for the *d*th dimension at a time t, r is randomly generated vectors in (0,1], β is the weight coefficient, T is the total number of iterations, $r_{i}$ is a random number between (0,1], and N is the search space dimension. The flowchart of the dFDB-MRFO-AAN algorithm is shown in Figure 5.

### 3.3. Overview of RUN-ANN Algorithm

RUNge Kutta optimizer (RUN) [45] is a novel swarm-based framework with stochastic elements. RUN’s main concept is based on the Runge Kutta (RK) method’s calculated slope, which is given by Equation (6), where y is a function of x [46].

(6) $$\begin{array}{l} y(x+\Delta x)=y(x)+\frac{1}{6}(c_{1}+2\times c_{2}+3\times c_{3}+c_{4})\Delta x\\ c_{1}=\frac{dy}{dx}=f(x,y)\;,\;c_{2}=f(x+\frac{\Delta x}{2},y+\frac{\Delta x}{2}\times k_{1})\;,\;\Delta x=x_{n+1}-x_{n}\\ c_{3}=f(x+\frac{\Delta x}{2}+y+\frac{\Delta x}{2}\times k_{2})\;,\;c_{4}=f(x+\Delta x,y+\Delta x\times k_{3}) \end{array}$$

**Figure 6:**
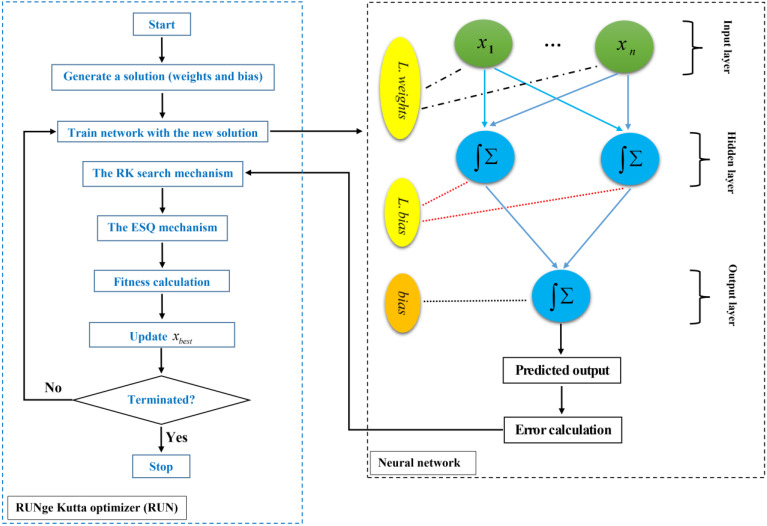
Flowchart of the RUN-ANN algorithm.

In the first step, for a population of size N, N positions are produced at random. Each member ($x_{n}$) in the population is a D-dimensional solution to an optimization problem. The RK technique is used by solutions to update their positions at each iteration (Equation (7)).

(7) $$\begin{array}{l} if\;rand<0.5\\ \;x_{n+1}=x_{c}+(2\cdot (0.5-rand)\cdot f)\cdot \frac{1}{6}\cdot (c_{1}+2\cdot c_{2}+2\cdot c_{3}+c_{4})\cdot \Delta x+\mu \cdot x_{s}\\ else\\ \;x_{n+1}=x_{m}+(2\cdot (0.5-rand)\cdot f)\cdot \frac{1}{6}\cdot (c_{1}+2\cdot c_{2}+2\cdot c_{3}+c_{4})\cdot \Delta x+\mu \cdot x_{s\prime }\\ end\\ \mu =0.5+0.1\cdot randn\;,\;f=a\cdot \exp(-b\cdot rand\cdot (\frac{i}{Max(i)}))\\ x_{s}=randn\cdot (x_{m}-x_{c})\;,\;x_{s\prime }=randn\cdot (x_{r1}-x_{r2})\\ x_{c}=\varphi \cdot x_{n}+(1-\varphi )\cdot x_{r1}\;,\;x_{m}=\varphi \cdot x_{b}+(1-\varphi )\cdot x_{lbest} \end{array}$$

where rand is a random value between 0 and 1, $x_{ri}$ is a random position, $x_{b}$ is the best position that achieved so far, $x_{lbest}$ is the best solution of the iteration, a and b are two constant numbers, i is iteration number, Max(i) is the total iteration number, and φ is a random number in (0,1]. In the RUN algorithm, enhanced solution quality (ESQ) is utilized to increase the solutions’ quality and prevent local optimum solutions in each algorithm iteration. In ESQ, the mean of three random choices and the best position are used to generate a new solution. The reader is directed to Ref. [45] for more information on the RUN method. The flowchart of the RUN-AAN algorithm is shown in Figure 6.

The mean absolute error (MAE, Equation (8)), the coefficient of determination (R^2^, Equation (9)), and coefficient of variation (C.O.V, Equation (10)) are utilized to assess the performance of hybrid algorithms.

(8) $$MAE=\frac{1}{n}\sum_{i=1}^{n}\left|y_{i}-{y_{i}}^{*}\right|$$

(9) $$R^{2}=1-\frac{\sum_{i=1}^{n}{\left(y_{i}-{y_{i}}^{*}\right)}^{2}}{\sum_{i=1}^{n}{\left(y_{i}-\bar{y}\right)}^{2}}$$

(10) $$C.O.V=\frac{\sigma }{\bar{y}}$$

where n presents the number of samples, $y_{i}$ is the recorded value, ${y_{i}}^{*}$ is the predicted value, $\bar{y}$ is the mean of the total of samples, and σ is standard deviation results.

## 4. Results and Discussion

**Figure 7:**
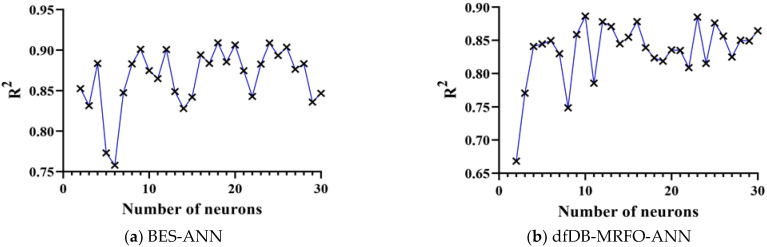

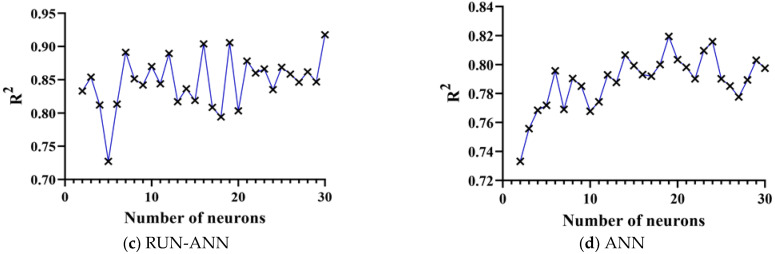
Changes in model performance versus changes in the number of neurons.

The neural network structure considered in this study has only three layers (input, hidden, and output layer). Parameters that must be determined by trial-and-error are the number of neurons in the hidden layer and the neural network activation function. ANNs with many neurons and functions in the middle layer are created and evaluated to discover the ideal structure for an ANN. Following the training and testing phases, networks with the Tansig function are selected for the middle and output layers (codes written in Matlab [47]). In addition, the number of neurons in the neural network’s hidden layer is increased from two to 30. Changes in the number of neurons in the inner layer are significant because they show the interaction between the input parameters. This means that increasing the number of neurons and creating more relationships if these relationships are not appropriate may reduce the efficiency of the neural network and the accuracy of network prediction. Figure 7 shows changes in models’ performance (coefficient of determination-$R^{2}$) versus changes in the number of neurons for different hybrid models. The maximum value of $R^{2}$ for BES-ANN, dfDB-MRFO-ANN, and RUN-ANN is 0.91, 0.88, and 0.92 with 18, 10, and 30 neurons, respectively. Figure 7d shows the results of the feed-forward neural network trained using backpropagation. All hybrid models outperform the feed-forward neural network trained using backpropagation.

**Figure 8:**
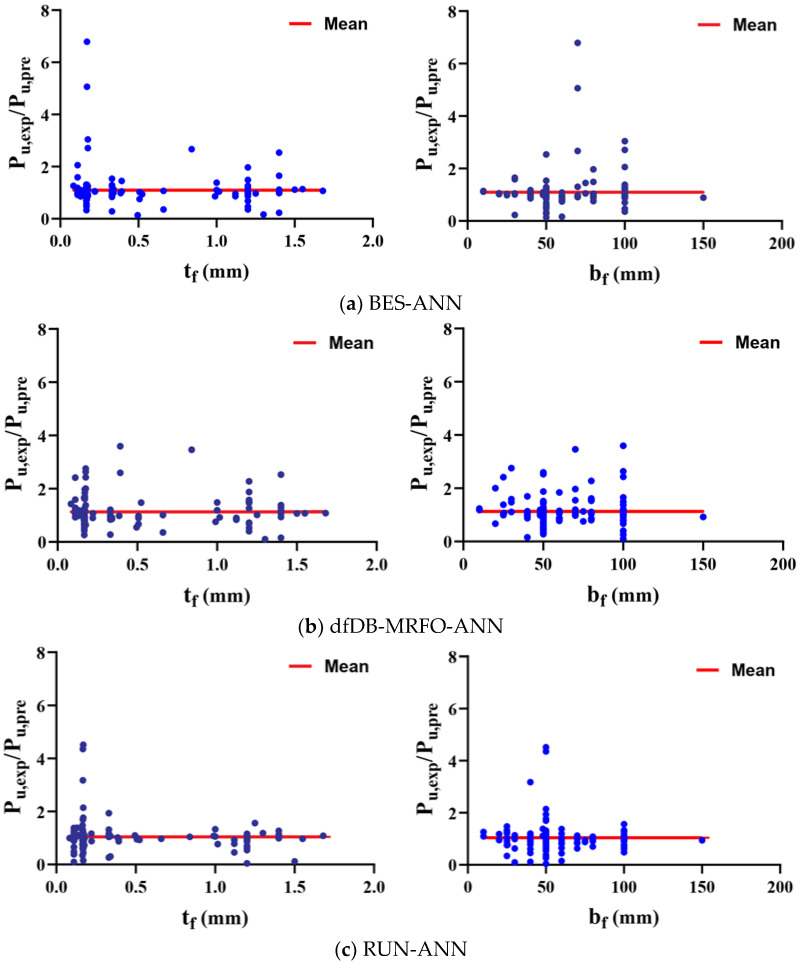
Variability in predicting with respect to the FRPS thickness.

Figure 8 shows the experimentally measured ($P_{u,\exp}$) to analytically-predicted ($P_{u,pre}$) ratio of the interfacial bond strength as a function of the FRPS thickness ($t_{f}$) and the FRPS width ($b_{f}$). Figure 8 depicts how hybrid approaches suffer from variability when estimating the effect of FRPS thickness on interfacial bond strength. Figure 8 also reveals that the novel RUN-ANN model, on the other hand, minimizes such predictions by reducing diversity. This enhanced performance is further validated by monitoring the impact of FRPS width ($b_{f}$). Table 2 summarizes the average of the models’ errors. The standard deviation is a number that expresses how far the values are spread out. A low standard deviation indicates that the majority of the data points are near to the average. Therefore, the coefficient of variation (C.O.V) also is computed. C.O.V represents the ratio of the standard deviation to the mean, and it is a useful statistic for comparing the degree of variation from one data series to another [20]. The mean of the RUN-ANN algorithm’s errors ($P_{u,\exp}/P_{u,pre}$) is quite near to a value of 1. The RUN-ANN also has the lowest standard deviation. The quantitative measures of the hybrid models indicate that the RUN-ANN algorithm is more in line with the experimental data. The value of standard deviation and C.O.V are high for the BES-ANN and dfDB-MRFO-ANN models, which means that there is a large amount of variability among the data points ($P_{u,\exp}/P_{u,pre}$). The RUN-ANN algorithm also has the lowest MAE (0.078), whereas MAE is 0.084 and 0.098 for the BES-ANN and dfDB-MRFO-ANN models.

**Figure 9:**
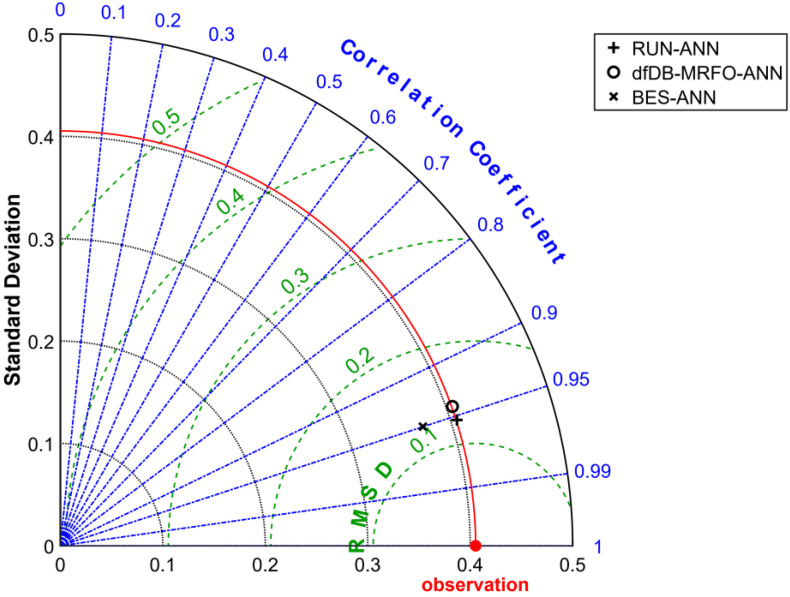
Taylor diagram of hybrid models.

Figure 9 shows the Taylor diagram of the hybrid models. Taylor diagrams, which are based on the Pearson correlation coefficient, the root-mean-square (RMS) error, and the standard deviation, are statistical diagrams that reveal which of the various models is the most reliable [48,49]. The more accurate a model predicts, the closer it is to the “observed” point. The standard deviation of models that lie on the solid red-arc is right (closer to the pattern of experimental data). In Figure 9 it can be seen that the RUN-ANN and dfDB-MRFO-ANN algorithms’ standard deviations are closer to the experimental data’s standard deviation. However, the RUN-ANN algorithm has a slightly higher correlation coefficient with experimental data. Although the RUN-ANN and BES-ANN models are the ones that most closely match experimental data (their points are near the point that shows as “observed” on the *x*-axis), the BES-ANN model’s standard deviation is smaller than the experimental data’s standard deviation.

### 4.1. Model Interpretations

For engineers and researchers, model interpretability is critical. As a result, engineers and researchers would like to discover how the model makes decisions and how the attributes influence the model’s outcome. SHapley Additive Explanation (SHAP) is utilized in this section to assist with model interpretation. Game theory’s Shapley value inspired the concept of the SHAPE method. The SHAP creates the EM() explanation model, which may be stated as:

(11) $$EM(z^{\prime })=\phi_{0}+\sum_{j=1}^{IF}\phi_{j}z_{j}^{\prime }$$

where $z^{\prime }\in {\left\{0,1\right\}}^{IF}$ IF is the number of input parameters, $\phi_{j}\in \mathbb{R}$ is the SHAP value for the *j*th input parameter, and $\phi_{0}$ stands for the baseline prediction, which is defined as all the features missing from the input space. The Shapley value specifies how the contributions should be distributed adequately among the attributes. The SHAP value for the *j*th input parameter can be determined using the following formula:

(12) ϕj=∑D⊆F{mi}D!(n−D−1)!n!v(D∪{mi})−v(D)D⊆{m1,m2,…,mn}mi , F={m1,m2,…,mn}

where $m^{i}$ is an instance, $\left|D\right|$ is the number of non-zero members of D, F{mi} represents the possible subsets that do not include $m^{i}$, and the model outcome for the subset D is represented by v(D). According to Equation (12), the model must be retrained twice for each subset: once for the subset including $m^{i}$ and once without it. The marginal contribution per variable is derived by multiplying the likelihood of occurrence for each subgroup without that variable by the different output with/without that variable [33].

**Figure 10:**
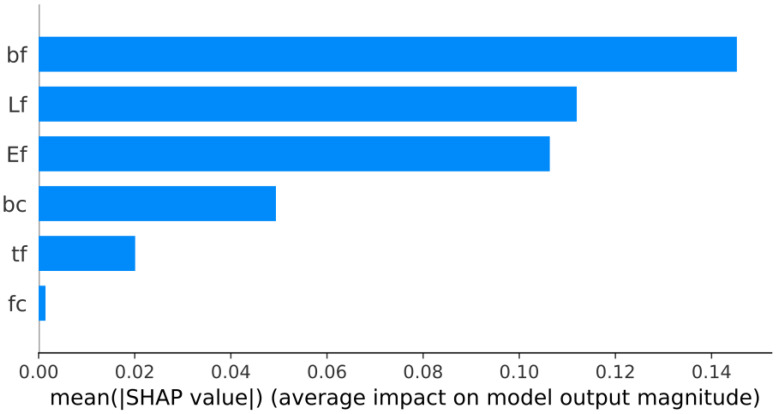
Bar-important plot of the best model.

**Figure 11:**
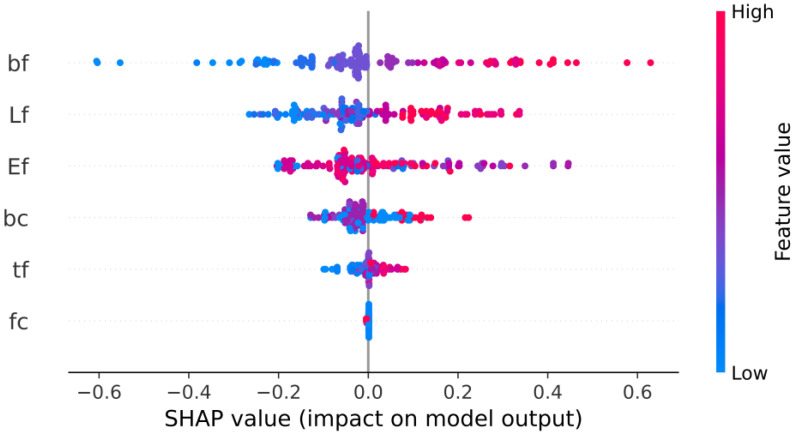
Summary plot.

Figure 10 shows the feature importance based on the best hybrid model (RUN-ANN). The parameters are listed in descending order on a bar-important plot based on their contribution. The top parameters have a higher predictive influence than the bottom ones, contributing more to the model prediction. In fact, the importance factor values in Figure 10 are the mean of the absolute SHAP values for features across the whole dataset. The most important variable is the FRPS width ($b_{f}$), which is followed by the bond length of the FRPS ($l_{f}$), FRPS elastic modulus ($E_{f}$), and width of the concrete ($b_{c}$). The SHAP approach can also illustrate how much each attribute impacts the target parameter, both positively and negatively, using the summary plot. The summary plot (Figure 11) is constructed from all of the samples in the test data. Figure 11 shows that $b_{f}$ and $l_{f}$ are positively correlated to the interfacial bond strength. In other words, increasing $b_{f}$ and $l_{f}$ leads to an increase in the bond strength.

### 4.2. Comparison with Mechanics-Based Models

It is useful to compare the RUN-ANN to existing equations. For comparisons, three equations from the literature are used, which are given as follows (the parameters are introduced in Section 2):

- Model 1: Wu et al. [50]

(13) $$\begin{array}{l} P_{u}=\left\{\begin{array}{l} 0.585b_{f}f_{c}^{0.1}k_{w}(E_{f}t_{f}{)}^{0.54}\;if\;l_{f}\geq l_{e}\\ 0.585b_{f}f_{c}^{0.1}k_{w}(E_{f}t_{f}{)}^{0.54}{\left(\frac{l_{f}}{l_{e}}\right)}^{1.2}\;if\;l_{f}<l_{e}\; \end{array}\right.\\ l_{e}=0.395(E_{f}t_{f}{)}^{0.54}/f_{c}^{0.09}\;,\;k_{w}=\sqrt{\frac{2.25-b_{f}/b_{c}}{1.25+b_{f}/b_{c}}} \end{array}$$

- Model 2: Lin et al. [12]

(14) $$\begin{array}{l} P_{u}=\frac{\alpha }{\beta }E_{f}t_{f}b_{f}k_{L}\;,\;\alpha =0.094f_{c}^{0.026}\;,\;\beta =\frac{0.134{\left(E_{f}t_{f}\right)}^{0.5}}{k_{w}f_{c}^{0.082}\;}\\ k_{L}=\frac{\eta \sqrt{1-\eta^{2}}\sinh(\sqrt{1-\eta^{2}}L/\beta )}{1+\eta \cosh(\sqrt{1-\eta^{2}}L/\beta )}\\ k_{w}=1+f_{c}^{0.385}[8(E_{f}t_{f}{)}^{-0.438}+0.001]\frac{{\left(1-b_{f}/b_{c}\right)}^{0.5}}{1+0.01b_{f}^{1.7}}\\ \eta =-3.61\times e^{-0.4454\frac{l_{f}}{\beta }}+4.11\times e^{-0.3835\frac{l_{f}}{\beta }} \end{array}$$

- Model 3: Wu et al. [51]

(15) $$\begin{array}{l} P_{u}=\beta_{w}b_{f}\sqrt{2(1+\frac{\lambda }{\sum })E_{f}t_{f}G_{cf}}\\ \beta_{w}=\sqrt{\frac{2-b_{f}/b_{c}}{1+b_{f}/b_{c}}}\;,\;\lambda =\frac{3.5}{t_{f}}\;,\;\sum =\frac{E_{f}}{4730\sqrt{f_{c}^{\prime }}}\;,\;G_{cf}=0.17 \end{array}$$

**Figure 12:**
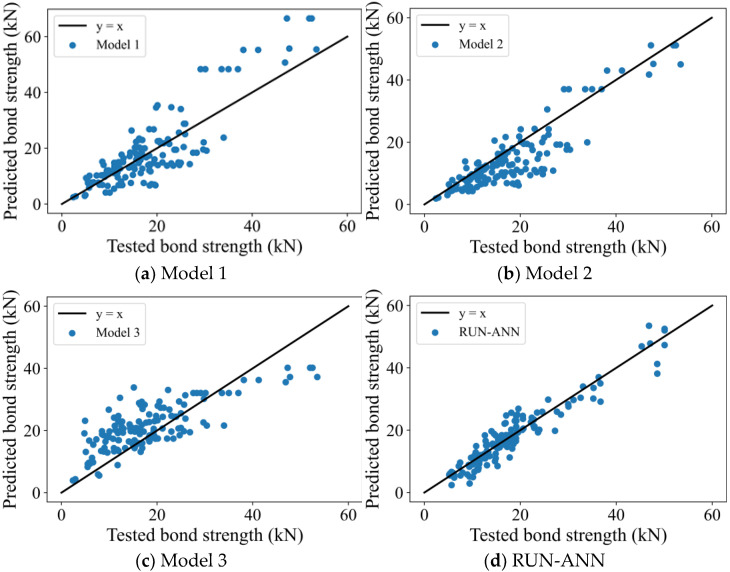
Comparisons of the Models.

The tested bond strength versus computed values from the three empirical models and the RUN-ANN is shown in Figure 12. Table 3 summarizes the quantitative measures of the experimentally measured ($P_{u,\exp}$) to analytically-predicted ($P_{u,pre}$) ratio for the models. It can be seen that selecting the best model from the three empirical models (Models 1–3) is difficult. The mean results of Model 1 and Model 2 are larger than 1, which reveals that the two models conservatively predict the bond strength. However, Model 3 overestimates the bond strength. The quantitative indicators of the RUN-ANN are notably better than that of the three empirical models. For example, the coefficient of variation of the tested to predict ratios from the empirical models is in the range of 34 to 43 percent, while for the RUN-ANN model, it will decrease to 18.6%. MAE of the RUN-ANN model is lower than 2.5 kN, while the MAE of the rest models is higher than 4 kN. These results demonstrate that the RUN-ANN model is more accurate than mechanics-based models.

The weakness of mechanics-based models can be due to the following reasons: (1) They were generated using a limited data set. As a result, when used to forecast experimental bond strength results, other than those utilized for calibration/validation, they show significant inconsistency and low accuracy. (2) Simple assumptions are considered in building these models because they are often constructed using standard mathematical programming and analytical or numerical methodologies. This makes complex nonlinear shear behaviors not well considered. In other words, despite their effectiveness in dealing with basic and idealized situations, similar strategies have exhibited significant flaws when dealing with complicated systems, primarily because of the simplifying assumptions that are taken into account when developing them.

### 4.3. Sensitivity Analysis Based on Sobol’s Method

**Figure 13:**
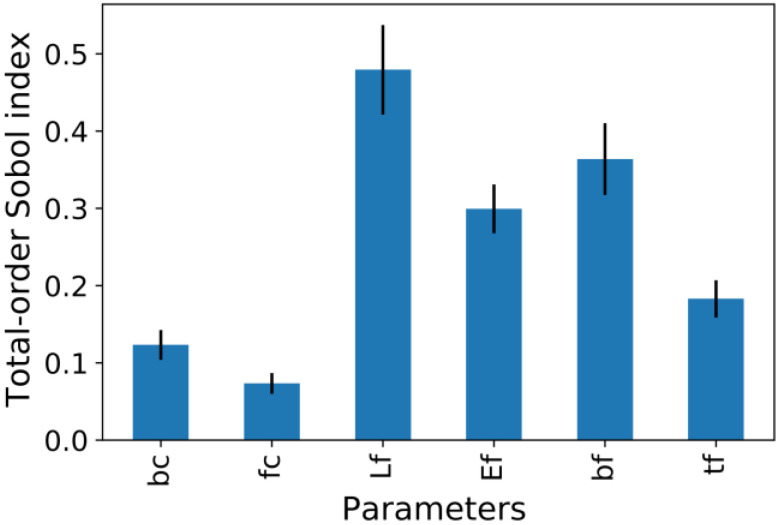
Sensitivity indices—total order index.

Another conventional method for investigating the impact of the changes in input parameters on the model’s outcome and vice versa is the use of the global sensitivity analysis (SA) based on Sobol’s method [52]. Compared with the local SA method suitable for linear models, the global SA technique is appropriate for complicated non-linear models [52]. The most prominent advantage of Sobol’s variance-based approach is that it does not require any special analytic functions and can be used in a variety of situations. The Python sensitivity analysis library (SALib [53]) is used in this study. A bar graph of the total order index is shown in Figure 13. Figure 13 indicates that the FRPS bond length ($l_{f}$), width ($b_{f}$), elastic modulus ($E_{f}$), and thickness ($t_{f}$) are the dominant parameters that contribute to the interfacial-bond strength. These results are in agreement with Chen et al. [27], which also reported that FRP sample’s width and bonding length were controlling factors in forecasting the interfacial-bond strength.

## 5. Conclusions

Fiber-reinforced polymers (FRPs) have been commonly used as an option to enhance reinforced concrete structures’ strength. The nonlinear relationships between the input variables and the bond strength make predicting the FRPC bond strength challenging. In this paper, novel hybrid approaches were developed for predicting the bond strength of FRPC using an artificial neural network (ANN) coupled with optimization algorithms, such as bald eagle search (BES), dynamic fitness distance balance (dFDB)-manta ray foraging optimization (MRFO) dFDB-MRFO, and RUNge Kutta optimizer (RUN). The weights and biases of the ANN model were adjusted using optimization algorithms. SHAP (SHapley Additive exPlanation) was used to determine the characteristics’ relevance.

The following conclusions were reached as a result of this research:

1. All hybrid models outperformed the feed-forward neural network trained using the backpropagation algorithm.
2. Among the studied hybrid models, the RUN-ANN algorithm was the most accurate model with the highest coefficient of determination (R^2^ = 92%), least mean absolute error (0.078), and least coefficient of variation (18.6%).
3. Compared with the mechanics-based models, the RUN-ANN model obtained the greatest prediction. The mean absolute error and coefficient of variation values of the RUN-ANN algorithm are 2.28 kN and 18.6%, lower than those of the three mechanics-based models.
4. Although the $R^{2}$ value of the BES-ANN and RUN-ANN was close, the BES-ANN model’s standard deviation, unlike the RUN-ANN model, was far from the experimental data’s standard deviation. In addition, the RUN-ANN model had a lower mean absolute error value.
5. Based on the SHAP method, the most important variable was the FRPS width ($b_{f}$), which was followed by the bond length of the FRPS ($l_{f}$), FRPS elastic modulus ($E_{f}$), and width of the concrete ($b_{c}$).
6. The global sensitivity analysis based on Sobol’s method indicated that the FRPS bond length ($l_{f}$), width ($b_{f}$), elastic modulus ($E_{f}$), and thickness ($t_{f}$) are the dominant parameters that contribute to the interfacial-bond strength.

## Figures and Tables

**Table 1:** Statistical properties of the dataset.

Statistical Information	Inputs						Output
	$b_{c}\;(mm)$	$f_{c}^{\prime }\;(MPa)$	$E_{f}\;(GPa)$	$t_{f}\;(mm)$	$b_{f}\;(mm)$	$l_{f}\;(mm)$	$P_{u}\;(kN)$
Mean	144.30	39.54	204.79	0.51	57.52	172.96	17.80
STD	57.63	15.21	78.14	0.57	26.39	101.02	10.39
Min	80	8	22.5	0.083	10	20	2.4
Max	500	75.5	425.1	4	150	400	56.5

**Table 2:** Characteristics of the predict-to-test ratio.

Model	BES-ANN	dfDB-MRFO-ANN	RUN-ANN
Mean	1.09	1.12	1.04
Standard deviation	0.68	0.52	0.19
C.O.V (%)	62.7	46.2	18.6
Median	0.99	1.02	0.98
MAE	0.084	0.098	0.078

**Table 3:** Model performance comparisons.

Model	Model 1	Model 2	Model 3	RUN-ANN
Mean	1.1	1.36	0.82	1.04
Standard deviation	0.47	0.48	0.28	0.19
Coefficient of variation (%)	42.7	35.5	34.0	18.6
Median	0.93	1.18	0.78	0.98
MAE (kN)	5.00	4.19	5.75	2.28

## Data Availability

All data are available in the paper. Additionally, data from this article can be found online at https://data.mendeley.com/datasets/hfk9syw9v5/1.
